# Utility of cardiac MRI in detecting diastolic dysfunction: comparison with Doppler echocardiography and tissue Doppler imaging

**DOI:** 10.1186/1532-429X-11-S1-P19

**Published:** 2009-01-28

**Authors:** Anthony F Tramontano, Cuilin Miao, Alison Hays, Robert Donnino, Ruth Lim, Leon Axel, Danny Kim, Hersh Chandarana, Monvadi B Srichai

**Affiliations:** NYU Medical Center, New York, NY USA

**Keywords:** Cardiac Magnetic Resonance, Diastolic Dysfunction, Diastolic Function, Deceleration Time, Tissue Doppler Imaging

## Background

Left ventricular diastolic dysfunction (LVDD) serves as a prognostic indicator of adverse cardiovascular events. Cardiac magnetic resonance (CMR) imaging is the gold standard for evaluating ventricular size and systolic function, but there is limited data on the utility of CMR in the evaluation of diastolic function. To assess the accuracy of CMR in assessing LVDD we compared quantitative CMR to Doppler echocardiography (ECHO).

## Methods

20 patients with cardiomyopathy underwent both CMR and ECHO studies. Through plane phase contrast CMR and Doppler ECHO was performed for assessment of early (E) and late (A) mitral inflow velocities, early mitral deceleration time (DT), and early (e') myocardial tissue velocities of the basal lateral wall. The two imaging methods were correlated for measurement of several parameters of diastolic function including E/A ratio, E DT, and e'. Diastolic function was assessed based on a combination of mitral inflow and myocardial tissue velocities and was classified as normal, abnormal relaxation, pseudo-normal or restrictive.

## Results

There was a statistically significant correlation for measurement of E/A ratio with both techniques (r = 0.827, p < 0.05), but not for any of the other parameters evaluated (E DT, e', E/e' ratio). Patients were classified by ECHO into diastolic functional grades as: 4 (20%) normal, 7 (35%) abnormal relaxation, 4 (20%) pseudo-normal, and 5 (25%) restrictive. Diastolic function grade was identical in 10 (50%) patients, differed by 1 grade in 5 (25%), differed by 2 grades in 3 (15%), and differed by 3 grades in 2 (10%). CMR tended to underestimate the degree of diastolic dysfunction in 70% of patients compared to ECHO, and there were 3 patients (15%) who were classified as normal by CMR, but who demonstrated some evidence of diastolic dysfunction on ECHO.

## Conclusion

CMR can detect the presence of LVDD with reasonable accuracy, although it tends to underestimate the degree when compared with Doppler ECHO.

